# Bias-Corrected Federated Learning for Video Recommendation over Stochastic Communication Links

**DOI:** 10.3390/e28040423

**Published:** 2026-04-09

**Authors:** Chaochen Zhou, Yadong Pei, Zhidu Li

**Affiliations:** 1 School of Communications and Information Engineering, Chongqing University of Posts and Telecommunications, Chongqing 400065, China; 2 Nanshan Sub-District Office, Nanan District, Chongqing 400065, China; 3 Key Laboratory of Public Big Data Security Technology, Chongqing College of Mobile Communication, Chongqing 401420, China

**Keywords:** federated learning, bias correction, statistical aggregation, video recommendation

## Abstract

With the increasing demand for privacy-preserving and real-time personalized services in large-scale video platforms, designing robust federated recommendation frameworks over practical communication networks has become increasingly important. To this end, this paper proposes a bias-corrected federated learning framework tailored for video recommendation over stochastic communication links. At the local training stage, a bias-corrected mechanism is introduced to explicitly account for video duration and user activity, mitigating feature-level bias and enabling the learned representations to more accurately reflect users’ intrinsic preferences. To meet the timeliness requirements of real-time federated learning, the successful upload probability of local model transmission is analytically characterized under time-varying channel conditions. Building upon this probabilistic model, a statistically corrected global aggregation strategy is designed to preserve the unbiasedness of the global update with respect to the ideal fully reliable FedAvg scheme, even when a subset of local nodes fails to upload their models within the specified delay constraint. Comprehensive experimental evaluations validate that the proposed framework significantly improves recommendation accuracy and maintains robustness against communication unreliability in practical distributed environments.

## 1. Introduction

With the rapid growth of video platforms, video recommendation systems have become a fundamental component for enhancing user experience and increasing platform engagement [1]. Video recommendation models rely heavily on large-scale user interaction data to capture personalized preferences and dynamically adapt to evolving interests [2]. Unlike traditional centralized scenarios, users in practical networks are naturally distributed across different geographical regions and access points, such as base stations or edge servers. Each edge node serves a group of users within its coverage area, and the composition of this user group is inherently dynamic due to user mobility, fluctuating network conditions, and time-varying activity patterns. As a result, the data distribution observed at each node continuously evolves over time. In such a distributed and dynamic environment, conventional centralized training is not only constrained by privacy concerns and communication overhead, but also struggles to promptly adapt to rapidly changing user preferences. Federated learning provides an effective solution by enabling distributed nodes to collaboratively train a global recommendation model without sharing raw user data [3]. Each node performs local training based on its currently served users and periodically uploads model updates to a central aggregator, thereby preserving user privacy while leveraging system-wide knowledge. However, the dynamic nature of user distribution further imposes stringent real-time requirements on federated video recommendation systems. Since the set of users associated with each node changes over time, the local data characteristics and preference patterns may shift accordingly. If the global model cannot be updated in a timely manner, it may fail to accurately reflect the latest user interests, leading to degraded recommendation performance. Therefore, ensuring timely and reliable model aggregation under distributed and time-varying user environments is a critical challenge for practical federated video recommendation systems [4].

However, enabling timeliness-aware federated learning in such a distributed and dynamic environment faces several critical challenges. On one hand, the heterogeneity of video content and user behavior may introduce significant bias into local model training. For example, intrinsic differences between long and short videos often lead to systematic discrepancies in user interaction patterns, such as watch time and completion ratio [5]. Similarly, highly active users typically generate substantially more interaction records than less active users, causing their preferences to dominate the local training objective [6]. When these factors are not properly addressed, the learned embeddings may encode duration-related or activity-related bias rather than genuine user interest. During federated aggregation, such biased local updates can be further amplified through iterative global optimization, ultimately degrading recommendation accuracy and fairness across different content types and user groups. On the other hand, the timeliness of model aggregation is strongly constrained by communication resources and channel conditions [7,8]. In practical edge networks, local nodes must upload model parameters to a central aggregator over stochastic communication links. The achievable transmission rate depends on factors such as allocated bandwidth, transmission power, interference, and channel fading. Under stringent delay constraints required by real-time recommendation, some nodes may fail to upload their local models within the prescribed deadline. Such stochastic upload failures not only slow down convergence, but may also introduce statistical bias into the aggregation process, since the global model is updated based on a random subset of participating nodes [9]. Consequently, a timeliness-aware federated video recommendation framework must jointly address two intertwined issues: mitigating training bias induced by heterogeneous content and user activity, and designing robust aggregation mechanisms that can cope with unreliable and resource-constrained communication links.

In the literature, substantial efforts have been devoted to incorporating classical recommendation models, such as FM [10], DeepFM [11], and Wide&Deep [12], into the federated learning framework [13,14,15]. Most of these studies primarily address the challenge of data heterogeneity under the standard FedAvg algorithm [16]. Optimization-based approaches, including FedProx [17] and SCAFFOLD [18], mitigate client drift by modifying the local objective function or introducing control variates. Personalized federated learning methods, such as pFedMe [19] and FedPAC [20], maintain client-specific model components to accommodate heterogeneous data distributions. Clustered federated learning frameworks, e.g., IFCA [21], partition clients into groups with similar statistical characteristics. Furthermore, knowledge distillation approaches (e.g., FedMD [22]) and contrastive learning methods such as MOON [23] enhance representation alignment under non-IID settings. Nevertheless, these works predominantly concentrate on mitigating statistical heterogeneity across clients, while largely overlooking two critical issues in real-time video recommendation scenarios. First, they do not explicitly address training bias introduced by intrinsic video attributes (e.g., duration) and user activity imbalance, which may distort preference modeling at the representation level. Second, they generally assume reliable and synchronous communication during global aggregation, without considering the timeliness constraints and stochastic upload failures caused by limited communication resources and time-varying wireless channels. Consequently, existing approaches may not be directly applicable to real-time federated video recommendation systems deployed over practical communication networks.

Motivated by this, this paper investigates a bias-corrected federated learning method for video recommendation over stochastic communication links. Specifically, video duration and user activity are explicitly incorporated into the feature modeling process to characterize content and user behavior more accurately, thereby mitigating the training bias induced by heterogeneous video attributes and activity imbalance. Furthermore, to capture the timeliness requirement of real-time federated learning, the successful upload probability of local model transmission is mathematically modeled and analyzed under stochastic wireless channels. Based on this probabilistic characterization, a statistically corrected global aggregation scheme is designed to ensure that the expected global update remains unbiased, even when some local nodes fail to upload their models within the prescribed delay threshold. Extensive experimental results demonstrate the effectiveness of the proposed framework in improving recommendation accuracy and maintaining robustness against communication unreliability. The main contributions of this paper can be summarized as follows:

- We propose a bias-corrected federated learning framework for video recommendation over stochastic communication links. By explicitly incorporating video duration and user activity into the feature modeling process, the proposed method effectively mitigates training bias caused by heterogeneous content attributes and user activity imbalance.
- We model and analyze the successful upload probability of local model transmission under stochastic wireless channels to capture the timeliness requirements of real-time federated learning. Based on this analysis, a statistically corrected global aggregation scheme is developed to guarantee that the expected global update remains unbiased, even in the presence of upload failures within the prescribed delay constraint.
- We conduct extensive experiments to validate the proposed framework. The results demonstrate that the proposed method improves recommendation accuracy while maintaining robustness against communication unreliability.

The remainder of this paper is organized as follows: Section 2 introduces the related works. Section 3 proposes a timeliness-aware federated video recommendation system. Section 4 introduces the bias-corrected local training method and statistical model aggregation method respectively. In Section 5, the experiment results are presented and analyzed. Section 6 finally concludes the paper.

## 2. Related Work

To fully exploit widely distributed user data while preserving privacy, federated learning has been extensively adopted for cooperative recommendation model training. For instance, in [24], a cross-institutional artwork similarity search and recommendation system was developed by integrating multimodal data fusion with federated learning. In [25], a privacy-preserving movie recommendation system was designed to ensure multi-level trustworthiness, where federated learning was employed to address non-IID local data and defend against input data reconstruction attacks. FedDeepFM was proposed in [14] as a federated deep recommendation model, in which model parameters rather than raw data are shared for global aggregation, and a pseudo-interaction filling strategy is introduced to prevent indirect inference attacks. Similarly, in [26], a federated recommendation framework based on attention-guided hierarchical reinforcement learning was constructed to identify critical user data while preserving privacy through coordinated aggregation. To further enhance federated learning performance, numerous studies have focused on mitigating data heterogeneity. FedProx [17] extends FedAvg by incorporating a proximal regularization term into the local objective to reduce client drift. SCAFFOLD [18] introduces control variates to correct local update bias and reduce variance caused by heterogeneous data distributions. Personalized federated learning approaches, such as pFedMe [19] and FedPAC [20], decouple global and local optimization or align feature representations while preserving client-specific components. Clustered federated learning methods like IFCA [21] partition clients into groups with similar data distributions to learn multiple global models. In addition, FedMD [22] leverages knowledge distillation over a shared public dataset to enable collaboration among heterogeneous client models, while MOON [23] adopts model-level contrastive learning to align local and global representations. Although these approaches address data heterogeneity from various optimization and architectural perspectives, they largely overlook the issue of feature construction in video recommendation scenarios. In particular, intrinsic video attributes such as duration, and user behavioral characteristics such as activity level may introduce systematic bias into representation learning. Without explicitly modeling and correcting such feature-level bias, the trained federated model may still suffer from performance degradation, even if statistical heterogeneity across clients is properly mitigated.

To mitigate feature bias that degrades recommendation performance, most existing studies primarily focus on video watch time or video duration. In [5], a causal graph was constructed to identify video duration as a confounding factor influencing both exposure and watch-time prediction, where the bias-inducing exposure effect was removed while preserving the intrinsic causal effect on watch time. In [27], a debiased multi-semantic labeling framework was proposed, in which quantile-based labels derived from the watch-time distribution emphasized relative ranking rather than absolute duration values, thereby better aligning model learning with ranking objectives. Quan et al. [28] introduced a video-length debiasing recommendation method to alleviate the tendency of models to favor longer videos in micro-video platforms. In [29], counterfactual watch time was modeled to estimate potential viewing duration unconstrained by video length, where user interest was inferred through a cost-based transformation and optimized via a counterfactual likelihood objective to address truncation bias. Additionally, ref. [30] proposed a user-aware popularity metric, termed personal popularity, to capture individualized item popularity based on users with similar interests. Although these approaches provide valuable insights into correcting duration-related or popularity-related bias, they predominantly concentrate on video-side features, particularly watch time or video length. To the best of our knowledge, limited attention has been paid to jointly modeling video attributes and user behavioral characteristics such as activity level in a unified framework to address feature level bias. The lack of a combined video-user debiasing mechanism may restrict the effectiveness of representation learning, especially in federated video recommendation scenarios where both content heterogeneity and user activity imbalance coexist.

Due to limited communication and computation resources, practical federated learning systems may not be able to support the participation of all local nodes in every global aggregation round. As a result, communication heterogeneity and stochastic transmission failures become critical factors affecting convergence performance. Existing studies addressing this issue can be broadly categorized into three main directions: leveraging historical gradients, optimizing client selection, and allocating wireless communication resources. To compensate for missing updates caused by transmission failures, several works exploit historical gradient information. For example, refs. [31,32] utilized previously received gradients to assist global aggregation when some local updates are unavailable. Similarly, ref. [33] shows that, under communication errors, convergence performance comparable to the error-free case can be achieved by reusing prior local updates for devices whose current transmissions are lost. Another line of research focuses on communication-aware resource optimization. In [34], an iterative algorithm was developed to jointly optimize time allocation, bandwidth allocation, transmit power, computational frequency, and learning accuracy, thereby mitigating communication-induced heterogeneity. Ref. [35] quantitatively analyzed the impact of wireless communication factors on federated learning performance and derives the optimal transmit power for each user based on the expected convergence rate under given client selection and resource block allocation strategies. In addition, ref. [36] derived analytical expressions for the convergence rate of federated learning in wireless environments by incorporating scheduling policies and inter-cell interference, and compares multiple client scheduling schemes. Furthermore, robust aggregation strategies have been investigated to explicitly model communication errors. In [37], packet error effects were incorporated into the aggregation process, and theoretical upper bounds on convergence are established for FL under non-convex settings. In [38], estimation errors in over-the-air computation are characterized via channel simulations, and a retransmission-based mechanism is proposed to improve model reliability over unreliable wireless links. Overall, while these studies provide important insights into handling communication heterogeneity and unreliable transmissions, they mainly focus on convergence analysis, client scheduling, or resource optimization. The interaction between stochastic communication timeliness and bias-sensitive recommendation tasks, particularly in federated video recommendation systems, remains relatively underexplored.

## 3. System Model

**Figure 1:**
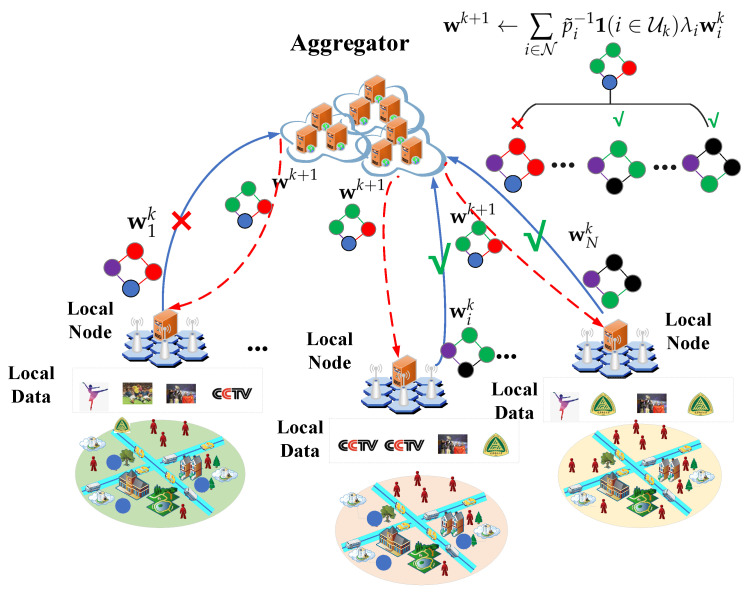
System model.

As depicted in Figure 1, the considered federated video recommendation system consists of one aggregator and multiple local nodes N={1,2,…,i,…,N}. Within the service area of each local node, the node collects historical user behavior data, analyzes user interests using recommendation algorithms, and delivers personalized video recommendations accordingly. However, due to the limited amount of locally available data, the performance of the recommendation algorithm may be constrained. To enhance model performance while preserving user privacy, it is beneficial to leverage user behavior data distributed across different local nodes. In this context, federated learning is introduced to enable collaborative training of the recommendation model without sharing raw data. The aggregator is responsible for aggregating model parameters uploaded from local nodes and coordinating the global model update process.

As users within each service area may change over time, the corresponding historical user behavior data can vary significantly across different periods. Therefore, to provide real-time and accurate recommendation services, it is essential to maintain model freshness during the federated learning process. In this regard, the model parameter upload process becomes critical. Since the communication links between each local node and the aggregator are time-varying, the transmission delays for model updates may differ across nodes, thereby affecting the timeliness and effectiveness of the global model update.

Below, the timeliness of model updates and the corresponding timeliness-aware federated learning process are modeled and analyzed.

### 3.1. Timeliness of Local Model Upload

For local node *i*, let $p_{i}$ denote the transmission power, $B_{i}$ the transmission bandwidth, $h_{i}$ the channel power gain, $\sigma_{i}^{2}$ the power of noise, and $I_{i}$ denote the interference power for the communication link. According to the Shannon Theorem, the achievable transmission rate of node *i* is

(1) $$\begin{array}{c} R_{i}=B_{i}\log_{2}\left(1+\frac{p_{i}h_{i}}{\sigma_{i}^{2}+I_{i}}\right) \end{array}$$

Accordingly, the upload delay for transmitting the local model is given by

(2) $$\begin{array}{c} t_{i}=\frac{L_{i}}{R_{i}}=\frac{L_{i}}{B_{i}\log_{2}\left(1+\frac{P_{i}h_{i}}{\sigma_{i}^{2}+I_{i}}\right)} \end{array}$$

where $L_{i}$ denotes the data size of local model, which can be quantified by experimental measurements.

Let ▵t denote the maximum tolerated delay for local model update, i.e., the local model of node *i* can be used for the current aggregation period when $t_{i}<▵t$. In this sense, the successful upload probability for node *i* holds as

(3) $$\begin{array}{c} Pr\left\{t_{i}<▵t\right\}=Pr\left\{\frac{L_{i}}{B_{i}\log_{2}\left(1+\frac{P_{i}h_{i}}{\sigma_{i}^{2}+I_{i}}\right)}<▵t\right\} \end{array}$$

Typically, in a given federated system with stochastic communication links, $L_{i}$, $p_{i}$, $B_{i}$ and $I_{i}$ are usually fixed and bounded, while the channel power gain $h_{i}$ is time-varying. Specifically, $h_{i}$ is commonly modeled as an independent and identically distributed random variable following standard channel models, such as Rayleigh fading or Rician fading. Moreover, since downlink communication resources are generally sufficient in practical systems, the transmission of the global model from the aggregator to local nodes is assumed to be always timely in this paper.

### 3.2. Timeliness-Aware Federated Learning

As a distributed machine learning paradigm, federated learning relies on a common model architecture shared across all local nodes. Each node performs local training based on its own dataset while maintaining the same global model structure. To facilitate mathematical analysis, let the dataset at node *i* be denoted by $D_{i}=\left\{1,2,\ldots ,m,\ldots ,D_{i}\right\}$. For each video data sample *m* at node *i*, the loss of the global model is defined as $f(w_{i},x_{i}^{m},y_{i}^{m})$, where w denotes the model parameter vector, $x_{i}^{m}$ is the input feature, and $y_{i}^{m}$ is the corresponding ground-truth label. Accordingly, the local loss function at node *i* is given by

(4) $$f_{i}\left(w\right)=\frac{1}{D_{i}}\sum_{m=1}^{D_{i}}f\left(w_{i},x_{i}^{m},y_{i}^{m}\right).$$

To evaluate the performance of the global model, the global loss function is defined as

(5) $$f\left(w\right):=\sum_{i\in N}\lambda_{i}f_{i}\left(w_{i}\right),$$

where $\lambda_{i}=\frac{D_{i}}{\sum_{i\in N}D_{i}}$ denotes the weight of node *i* on the global FL model. Consequently, the conventional optimization objective of federated learning can be formulated as the minimization of the global loss function, i.e.,

(6) $$\min_{w}f\left(w\right)=\sum_{i\in N}\lambda_{i}f_{i}\left(w_{i}\right).$$

With the aforementioned definitions, a generalized federated learning procedure, namely FedAvg, is summarized in Algorithm 1 [16]. In Step 7, the local update is performed under the assumption that the local loss function $F_{i}\left(w\right)$ is differentiable. In Step 10, the global model is updated by computing the weighted average of the parameters received from all local nodes. It is worth noting that Steps 7 and 10 are critical components of the FL algorithm, as they directly affect the final performance of the local and global models respectively. Since the timeliness of local model uploads is crucial for federated learning-based training of video recommendation algorithms, it is necessary to design improved parameter aggregation schemes that explicitly account for update freshness, thereby enhancing the robustness and effectiveness of the federated learning process.

Since some local nodes may fail to upload their trained models to the aggregator within the required time, the parameter aggregation in Step 10 of Algorithm 1 is performed only over the set of timely participating nodes. Accordingly, the global model update at round *k* can be expressed as

(7) $$w^{k+1}\leftarrow \sum_{i\in N}1\left(i\in U_{k}\right)\lambda_{i}w_{i}^{k},$$

where $U_{k}\subseteq N$ denotes the set of local nodes that successfully upload their model parameters to the aggregator within the deadline during the *k*-th aggregation round, and 1(·) is the indicator function.

**Figure 2:**
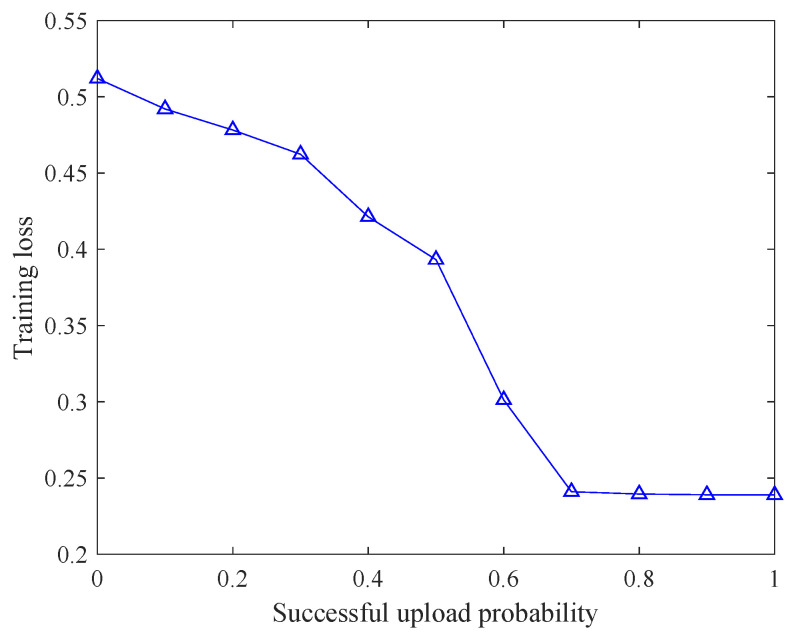
Training loss varying with successful upload probability.

According to (7), the aggregation weights of the participating local nodes differ from those in conventional federated learning. Such weight distortion, resulting from partial participation, may introduce bias into the global update and consequently degrade the overall model performance. Figure 2 depicts the variation of the training loss of FedAvg with respect to the successful upload probability. The results demonstrate that when some local nodes fail to upload their models to the aggregator within the prescribed time, the performance of the global model deteriorates accordingly.
**Algorithm 1** Traditional Federated Learning**Input:** Local learning rate η, aggregation times *K*, local update times τ **Output:** Model parameters $w^{f}$
1:Initialize w as a random matrix2:**for** k=0,1,...,K−1 **do**3:   Send $w^{k}$ to all local nodes4:   **for** i=1,2,...,N **do**5:       Initialize $w_{i}^{k}\leftarrow w^{k}$6:       **for** j=0,1,...,τ−1 **do**7:           $w_{i}^{k,j+1}\leftarrow w_{i}^{k,j}-\eta \nabla f_{i}\left(w_{i}^{k,j}\right)$, where $\nabla f_{i}\left(w_{i}^{k,j}\right)$ denotes the local gradient.8:       **end for**9:   **end for**10:   Update global parameter as $w^{k+1}\leftarrow \sum_{i\in N}\lambda_{i}w_{i}^{k}$11:**end for**12:$w^{f}\leftarrow w^{K}$

## 4. The Proposed Federated Learning Method

### 4.1. Bias-Corrected Local Training

Current video recommendation systems commonly adopt viewing duration as a primary indicator of user interest. However, longer videos inherently accumulate longer watch times, which introduces a duration bias; that is, the system tends to favor long videos over short ones in recommendation results. When neural network models are trained on such biased datasets via gradient descent, this bias can be further amplified during the loss minimization process. The underlying reasons are twofold: (1) the gradient direction of long-video embeddings is more likely to align with gradients induced by positive user interactions; and (2) the magnitude of positive gradients associated with long videos is typically larger than that of short videos. Consequently, the training dynamics progressively reinforce the preference for long videos, thereby exacerbating recommendation bias. To address this issue, we propose an embedding-based bias correction algorithm for local training at each node.

**Figure 3:**
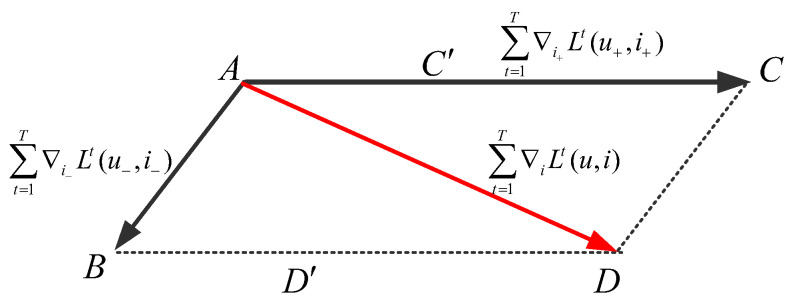
Impact of positive and negative sample gradients on combined gradients.

Let $\vec{AC}$ and $\vec{AB}$ denote the accumulated positive and negative gradients, respectively. As depicted in Figure 3, the overall gradient $\vec{AD}=\vec{AC}+\vec{AB}$ leans toward $\vec{AC}$ due to its larger magnitude, causing the learned video embeddings to primarily favor positive interactions. These accumulated gradients are obtained by summing gradients across all iterations during optimization of the objective function $L^{+}\left(s,m\right)$.

To mitigate this effect, we first normalize user embeddings to suppress the influence of highly active users and record the cumulative gradients of both user and video embeddings. And then the embeddings are further adjusted based on the accumulated gradient information to compensate for biases introduced by video duration and user conformity.

For the service area of node *i*, let S denote the set of users and M denote the set of videos. The user–video interaction matrix is defined as $Y\in {\left\{0,1\right\}}^{\left|S|\times |M\right|}$, where $Y_{sm}=1$ indicates that user s∈S has interacted with video m∈M.

The predicted rating function for user *s* and video *m* is defined as

(8) $${\hat{y}}_{sm}=P_{s}^{⊤}Q_{m},$$

where ${\hat{y}}_{sm}$ denotes the predicted score, $P_{s}$ represents the latent embedding vector of user *s*, and $Q_{m}$ denotes the latent embedding vector of video *m*.

For a learned recommendation system, it analyzes the interest preference of user *s* and generates a personalized ranked list $R_{s}$ of potentially preferred videos:

(9) $$\begin{array}{cc} R_{s} & =\;{\left\{\left(m,\mathrm{rank}({\hat{y}}_{sm})\right)\right\}}_{m\in M} \end{array}$$

(10) $$\begin{array}{cc} \; & =\;{\left\{\left(m,\mathrm{rank}(P_{s}^{⊤}Q_{m})\right)\right\}}_{m\in M} \end{array}$$

(11) $$\begin{array}{cc} \; & =\;\{{\left(m,\mathrm{rank}\left(∥P_{s}∥∥Q_{m}∥\cos\left(P_{s},Q_{m}\right)\right)\right\}}_{m\in M}, \end{array}$$

where rank(·) denotes the ranking function applied to the set of predicted scores between user *s* and all videos m∈M. As indicated by the above expression, the personalized ranking results depend on the magnitudes of the user and video embeddings as well as their cosine similarity.

Hence, we employ the Bayesian Personalized Ranking (BPR) loss to optimize the embedding model by comparing user preferences between positive and negative samples:

(12) $$L_{\mathrm{BPR}}=\frac{1}{|D_{a}|}\sum_{\left(s,m,v\right)\in D_{a}}\log\left(\sigma \left({\hat{y}}_{sm}-{\hat{y}}_{sv}\right)\right)-\frac{1}{2}\beta_{\Theta }{∥\Theta ∥}^{2}$$

where $D_{a}$ denotes the set of (s,m,v) triplets. Specifically, (s,m) represents a positive sample, corresponding to an observed interaction where the user explicitly favors the video, while (s,v) denotes a negative sample, representing an unobserved interaction. Here, Θ denotes the local model parameters, and σ(x)=1/(1+exp(−x)) is the sigmoid function. Accordingly, the gradient of the BPR loss with respect to the model parameters Θ is derived as

(13) $$\begin{array}{cc} \frac{\partial L_{\mathrm{BPR}}}{\partial \Theta } & =\;\frac{1}{|D_{a}|}\sum_{\left(s,m,v\right)\in D_{a}}\frac{\partial \log\sigma \left({\hat{y}}_{sm}-{\hat{y}}_{sv}\right)}{\partial \Theta }-\beta_{\Theta }\frac{\partial {∥\Theta ∥}^{2}}{\partial \Theta } \end{array}$$

(14) $$\begin{array}{cc} \; & =\;\frac{1}{|D_{a}|}\sum_{\left(s,m,v\right)\in D_{a}}\frac{-\left({\hat{y}}_{sm}-{\hat{y}}_{sv}\right)}{1+\exp^{-\left({\hat{y}}_{sm}-{\hat{y}}_{sv}\right)}}\cdot \frac{\partial \left({\hat{y}}_{sm}-{\hat{y}}_{sv}\right)}{\partial \Theta }-\beta_{\Theta }\Theta \end{array}$$

The gradient used to update the matrix factorization model is derived as follows:

(15) $$\frac{\partial \left({\hat{y}}_{sm}-{\hat{y}}_{sv}\right)}{\partial \Theta }=\left\{\begin{array}{cc} Q_{m}-Q_{v} & \mathrm{if}\;\Theta =P_{s},\\ P_{s} & \mathrm{if}\;\Theta =Q_{m},\\ -P_{s} & \mathrm{if}\;\Theta =Q_{v},\\ 0 & \mathrm{otherwise}. \end{array}\right.$$

For each user–video pair (s,m) with positive interaction, the model randomly samples a negative pair (s,v). Regarding the gradient with respect to the user feature vector $P_{s}$, if the features of the positive and negative samples are very similar, the gradient will approach zero, indicating limited room for adjusting the user representation. Conversely, if the positive and negative samples differ significantly, the gradient magnitude becomes larger, driving $P_{s}$ closer to the positive sample $Q_{m}$ while pushing it away from the negative sample $Q_{v}$.

For videos with positive interactions, the embedding vector $Q_{m}$ is updated to be more aligned with the user feature vector $P_{s}$. For videos with negative interactions, the embedding vector is updated to move farther away from the user feature vector. Through this gradient update mechanism, the distance between positive and negative sample embeddings increases, while ensuring that both types of sample pairs contribute equally to the overall gradient direction.

Thus, at the *t*-th iteration, the latent vector updates for the positive video embedding $Q_{m}^{t}$, the negative video embedding $Q_{v}^{t}$, and the user embedding $P_{s}^{t}$ are given as follows:

(16) $$\begin{array}{cc} Q_{m}^{t} & =\;Q_{m}^{t-1}-\mathrm{lr}\cdot P_{s}^{t-1}, \end{array}$$

(17) $$\begin{array}{cc} Q_{v}^{t} & =\;Q_{v}^{t-1}+\mathrm{lr}\cdot P_{s}^{t-1}, \end{array}$$

(18) $$\begin{array}{cc} \;P_{s}^{t} & =\;P_{s}^{t-1}-\mathrm{lr}\cdot \left(Q_{s}^{t-1}-Q_{v}^{t-1}\right), \end{array}$$

where lr denotes the learning rate. The above expressions indicate that video embeddings and user embeddings mutually influence each other during training. Let $Q_{m}^{0}$ denote the initial latent vector of video *m*. According to the update rule, the equivalent form of $Q_{m}^{t}$ can be expressed as

(19) $$Q_{m}^{t}=Q_{m}^{0}-\mathrm{lr}\cdot \left(\sum_{\left(s,m\right)\in Y_{m}^{+,t-1}}P_{s}-\sum_{\left(s^{\prime },m\right)\in Y_{m}^{-,t-1}}P_{s^{\prime }}\right),$$

where $Y_{m}^{+,t-1}$ and $Y_{m}^{-,t-1}$ denote the sets of positive and negative user samples associated with video *m* before the *t*-th iteration, respectively.

Let $\Delta_{m}$ denote the cumulative gradient of $Q_{m}$, defined as

(20) $$\Delta_{m}=\sum_{\left(s,m\right)\in Y_{m}^{+}}P_{s}-\sum_{\left(s^{\prime },m\right)\in Y_{m}^{-}}P_{s^{\prime }}.$$

Since the users in $Y_{i}^{+}$ and $Y_{i}^{-}$ consist of both active and inactive users, Equation (20) can be further extended as

(21) $$\begin{array}{cc} \Delta_{m}\; & =\sum_{\left(s,m\right)\in Y_{m,act}^{+}}P_{s}+\sum_{\left(s,m\right)\in Y_{m,inact}^{+}}P_{s}\\ \; & \;-\sum_{\left(s^{\prime },m\right)\in Y_{s,act}^{-}}P_{s^{\prime }}-\sum_{\left(s^{\prime },i\right)\in Y_{m,inact}^{-}}P_{s^{\prime }} \end{array}$$

Similarly, we have

(22) $$\begin{array}{c} \Delta_{s}=\sum_{\left(s,m\right)\in Y_{s,long}^{+}}Q_{m}+\sum_{\left(s,m\right)\in Y_{s,short}^{+}}Q_{m}\;-\sum_{\left(s,v\right)\in Y_{s,long}^{-}}Q_{v}-\sum_{\left(s,vj\right)\in Y_{s,short}^{-}}Q_{v} \end{array}$$

Here, long denotes videos with relatively long durations, while short denotes videos with relatively short durations.

Therefore, based on Equations (21) and (22), the imbalance in the accumulated positive and negative gradients can be attributed to the following factors: (1) differences in the frequency of user–video interactions; (2) variations in video duration; and (3) conformity effects arising from users interacting alongside other active users. Hence, it is necessary to disentangle them and apply differentiated treatments in order to mitigate their impact.

Based on the preceding analysis, video duration is a primary factor contributing to the imbalance between accumulated positive and negative gradients. To eliminate this duration-induced bias, the video embedding is decomposed as

(23) $$Q_{m}=Q_{m}^{\mathrm{time}}+Q_{m}^{\mathrm{int}},$$

where $Q_{m}^{\mathrm{time}}$ captures the effect of video duration, and $Q_{m}^{\mathrm{int}}$ represents the intrinsic content features of the video.

Similarly, the user embedding is formulated as

(24) $$P_{s}={∥P_{s}^{\mathrm{act}}∥}_{2}\left(P_{s}^{\mathrm{conf}}+P_{s}^{\mathrm{int}}\right),$$

where $P_{s}^{\mathrm{act}}$ characterizes the user’s activity level, $P_{s}^{\mathrm{conf}}$ denotes the user’s conformity to mainstream preferences, and $P_{s}^{\mathrm{int}}$ represents the user’s genuine interests. Since highly active users typically generate more interactions, a multiplicative formulation is adopted to model the scaling effect of user activity.

Typically, the unbiased matching score between user *s* and video *m* should depend only on their intrinsic components:

(25) $${\hat{y}}_{sm}=P_{s}^{\mathrm{int}}\cdot Q_{m}^{\mathrm{int}}.$$

Highly active users tend to accumulate larger gradients due to their extensive interaction histories. For example, consider two users $s_{1}$ and $s_{2}$ with similar intrinsic interests but different activity levels. Although their embedding update directions are similar, the norm of $s_{1}$’s embedding becomes significantly larger. This increased magnitude amplifies the gradient updates of associated video embeddings, thereby aggravating the imbalance between positive and negative gradients. To mitigate the dominant influence of highly active users on the aggregated gradient direction, each user embedding is normalized to a unit vector during training:

(26) $${\hat{P}}_{s}=\frac{P_{s}}{{∥P_{s}^{\mathrm{act}}∥}_{2}}.$$

**Figure 4:**
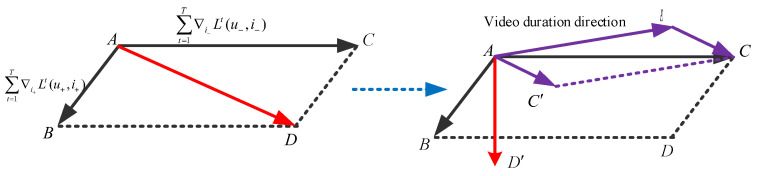
Bias corrected method.

To further alleviate the bias introduced by video duration, we correct the duration bias by accumulating and compensating for the duration-related component in the positive gradients. As illustrated in Figure 4, by adjusting the accumulated positive gradient direction $\vec{AC}$, the biased aggregated gradient direction shifts from $\vec{AD}$ to a debiased direction $\vec{AD^{\prime }}$.

A key issue is how to approximate $Q_{m}^{\mathrm{time}}$ in (23). In this paper, the average embedding of long-duration videos can be used to approximate the duration bias component $Q^{\mathrm{time}}$, and the corresponding bias direction is denoted by $\bar{Q}$. Therefore, by decomposing the video embedding $Q_{m}$ into its intrinsic component $Q_{m}^{\mathrm{int}}$ and its projection onto the duration-bias direction, the intrinsic video representation can be obtained by removing the projected component:

(27) $$Q_{m}^{\mathrm{int}}=Q_{m}-Q_{m}^{\mathrm{time}}=Q_{m}-\alpha_{1}\cdot \cos\;\left(Q_{m},\bar{Q}\right)\cdot {∥Q_{m}∥}_{2}\cdot \bar{Q},$$

where $\alpha_{1}$ is a scaling coefficient that controls the strength of duration-bias removal.

Similarly, the intrinsic interest component of user *u* can be derived as

(28) $$P_{s}^{\mathrm{int}}={\hat{P}}_{s}-\alpha_{2}\cdot \cos\;\left({\hat{P}}_{s},\bar{P}\right)\cdot {∥{\hat{P}}_{s}∥}_{2}\cdot \bar{P},$$

where $\alpha_{2}$ is the corresponding scaling coefficient, and $\bar{P}$ represents the average conformity-bias direction in the user embedding space.

Algorithm 2 summarizes the proposed bias-corrected method. Specifically, Steps 1–9 perform BPR-based iterative representation learning, in which user–item interaction pairs are sampled, embeddings are generated and normalized, pairwise matching scores are computed, and model parameters are optimized using the BPR loss. Through this process, the learned embeddings gradually encode both personalized preference signals and latent bias components. After convergence, Step 11 computes the averaged user and item embeddings to estimate the global bias direction in the feature space. Finally, Steps 12–13 remove the bias component from each embedding by subtracting its projection onto the corresponding global direction based on cosine similarity, thereby yielding bias-corrected user and item representations.

Based on the obtained intrinsic feature vectors $Q_{m}^{\mathrm{int}}$ and $P_{s}^{\mathrm{int}}$, the input to the local training model is constructed as

(29) $$x_{i}=\left[Q_{m}^{\mathrm{int}}\;∥\;P_{s}^{\mathrm{int}}\right],$$

where ∥ denotes the concatenation operation.

Subsequently, recommendation models such as DeepFM, Wide&Deep, and other neural architectures can be employed, together with appropriate loss functions (e.g., the cross-entropy loss), to optimize the local objective. Through this training process, the local model parameters $w_{i}^{k}$ at the *k*-th aggregation round are obtained.
**Algorithm 2** Bias-Corrected Method**Input:** User embedding matrix P, video embedding matrix Q, interaction matrix Y, initial embedding model Θ, learning rate lr, bias-correction parameters $\alpha_{1}$ and $\alpha_{2}$ **Output:** Debiased user embeddings $P^{\mathrm{int}}$ and debiased video embeddings $Q^{\mathrm{int}}$
1:Initialize Θ as a random matrix2:**for** t=1,2,…,T **do**3:    Sample a mini-batch of user–item interactions from $Y^{t}$, construct positive set $Y^{+,t}$ and negative set $Y^{-,t}$4:    Initialize the user and video embedding vectors $\left\{{\tilde{P}}_{s},{\tilde{Q}}_{m},{\tilde{Q}}_{v}\right\}=f_{\Theta }\left(\Theta \right)$5:    Normalized user embedding vector ${\hat{P}}_{s}\leftarrow P_{s}/{∥P_{s}∥}_{2}$6:    Update the embedding vector according to Equations (16)–(18)7:    Compute sample matching score set $\left\{{\hat{y}}_{sm},{\hat{y}}_{sv}\right\}$, where ${\hat{y}}_{sm}={\hat{P}}_{s}^{T}{\tilde{Q}}_{m}$ and ${\hat{y}}_{sv}={\hat{P}}_{s}^{T}{\tilde{Q}}_{v}$8:    Compute BPR loss $L_{\mathrm{BPR}}=\frac{1}{|Y^{t}|}\sum_{\left(s,m,v\right)\in Y^{t}}\log\left(\sigma \left({\hat{y}}_{sm}-{\hat{y}}_{sv}\right)\right)-\frac{1}{2}\beta_{\Theta }{∥\Theta ∥}^{2}$9:    Update the parameters $\Theta =\Theta -\mathrm{lr}\nabla L_{\mathrm{BPR}}$10:**end for**11:Average $\tilde{P},\tilde{Q}$ to obtain $\bar{P},\bar{Q}$ respectively12:Compute unbiased user embedded vector $P_{s}^{\mathrm{int}}={\hat{P}}_{s}-\alpha_{2}\cdot \cos\;\left({\hat{P}}_{s},\bar{P}\right)\cdot {∥{\hat{P}}_{s}∥}_{2}\cdot \bar{P}$13:Compute unbiased video embedded vector $Q_{m}^{\mathrm{int}}=Q_{m}-\alpha_{1}\cdot \cos\;\left(\bar{Q},Q_{m}\right)\cdot {∥Q_{m}∥}_{2}\cdot \bar{Q}$

### 4.2. Statistical Global Aggregation

In this subsection, we aim to mitigate the adverse impact of unsuccessful model uploads on parameter aggregation, thereby improving the overall performance of the federated learning model. We first revisit the aggregation process in (7), which naturally extends the conventional federated learning aggregation rule to scenarios with unreliable model uploads. However, the reassignment of aggregation weights may be negatively affected by the successful upload probability, leading to instability in the global update.

To address this issue, we first introduce the notion of aggregation stability and then propose an improved aggregation scheme to enhance robustness.

Let ${\tilde{w}}^{k}:=\sum_{i\in N}\lambda_{i}w_{i}^{k-1}$ denote the ideal global model parameters after *k* aggregation rounds under perfectly reliable uploads. Let $w^{k}\left(G_{1}\right)$ and $w^{k}\left(G_{2}\right)$ denote the global parameters obtained under aggregation policies $G_{1}$ and $G_{2}$, respectively. The stability of aggregation is defined as follows.

**Definition (Aggregation Stability):** The FL scheme $G_{2}$ is said to be stabler than $G_{1}$ if

(30) $$\underset{k\in \{0,1,\ldots ,K\}}{\sup}E∥w^{k}\left(G_{1}\right)-{\tilde{w}}^{k}∥\geq \underset{k\in \{0,1,\ldots ,K\}}{\sup}E∥w^{k}\left(G_{2}\right)-{\tilde{w}}^{k}∥.$$

The intuition is to compare the deviation between the aggregated parameters and the ideal global parameters at each round. A smaller deviation implies that the aggregation scheme produces updates closer to the ideal case, thereby ensuring a more stable FL process.

To reduce model deviation, we propose a statistically corrected aggregation scheme:

(31) $$w^{k+1}\leftarrow \sum_{i\in N}P_{i}^{-1}1\left(i\in U_{k}\right)\lambda_{i}w_{i}^{k},$$

where $P_{i}=E\left[1\left(i\in U_{k}\right)\right]$ denotes the successful upload probability of node *i*. Since the event $i\in U_{k}$ indicates that node *i* successfully uploads its local model in round *k*, we have

(32) $$P_{i}=\mathrm{Pr}\left\{t_{i}<\Delta t\right\}=\mathrm{Pr}\;\left\{\frac{L_{i}}{B_{i}\log_{2}\;\left(1+\frac{P_{i}h_{i}}{\sigma_{i}^{2}+I_{i}}\right)}<\Delta t\right\}.$$

Taking expectation on both sides of (31), we obtain

(33) $$E\left[w^{k+1}\right]=\sum_{i\in N}P_{i}^{-1}E\left[1\left(i\in U_{k}\right)\right]\lambda_{i}w_{i}^{k}=\sum_{i\in N}\lambda_{i}w_{i}^{k}.$$

Therefore, the proposed aggregation scheme is statistically unbiased with respect to the ideal FL scheme where all local models upload successfully.

In contrast, for the conventional aggregation scheme in (7), we have

(34) $$E\left[w^{k+1}\right]=\sum_{i\in N}P_{i}\lambda_{i}w_{i}^{k},$$

which deviates from the ideal aggregation unless $P_{i}=1$ for all *i*.

According to (30), the proposed aggregation rule achieves a smaller deviation from the ideal model in expectation, and thus yields performance closer to the optimal case compared with the conventional aggregation scheme.

In summary, the proposed bias-corrected federated learning framework over stochastic communications is presented in Algorithm 3. Compared with classical federated learning, the proposed approach introduces two key modifications: First, prior to local model training, the input features at each client are debiased using the bias-correction procedure described in Algorithm 2 (Steps 1–3), ensuring that local updates are performed on bias-reduced representations. Second, the global aggregation rule is revised in Step 13 to account for stochastic communication effects, where the server updates the global model using a statistically corrected weighted aggregation scheme rather than standard FedAvg. Through the integration of feature-level bias correction and communication-aware aggregation, the proposed method enhances robustness and stability under heterogeneous data distributions and unreliable communication conditions.
**Algorithm 3** Bias-Corrected Federated Learning over Stochastic Communications**Input:** Local learning rate η, aggregation times *K*, local update times τ **Output:** Model parameters $w^{f}$
1:**for** i=1,2,...,N **do**2:    Applied Algorithm 2 to obtain the local training feature for node *i*3:**end for**4:Initialize w as a random matrix5:**for** k=0,1,...,K−1 **do**6:    Send $w^{k}$ to all local nodes7:    **for** i=1,2,...,N **do**8:        Initialize $w_{i}^{k}\leftarrow w^{k}$9:        **for** j=0,1,...,τ−1 **do**10:            $w_{i}^{k,j+1}\leftarrow w_{i}^{k,j}-\eta \nabla f_{i}\left(w_{i}^{k,j}\right)$, where $\nabla f_{i}\left(w_{i}^{k,j}\right)$ denotes the local gradient.11:        **end for**12:    **end for**13:    Update global parameter as $w^{k+1}\leftarrow \sum_{i\in N}P_{i}^{-1}1\left(i\in U_{k}\right)\lambda_{i}w_{i}^{k}$14:**end for**15:$w^{f}\leftarrow w^{K}$


## 5. Results and Analysis

### 5.1. Experiment Setup

#### 5.1.1. Basic Settings

Unless otherwise specified, the basic experimental settings are as follows: The number of local nodes is set to 10. For each node, the transmission power for model uploading is set to 1 W, the allocated bandwidth is 100 kHz, the interference power is $10^{-5}$ W, the noise power is $10^{-9}$ W, and the maximum upload delay is 3 s. The classic Rayleigh channel model is employed to simulated the local model upload process, where the channel power gain between each node and the aggregator is assumed to follow an exponential distribution with an average value of 1.

The federated learning process consists of 100 aggregation rounds. In each round, every local node performs 10 local training epochs before participating in global model aggregation. The batch size is configured as 2048, and the DeepFM architecture is adopted as the base recommendation model for local training. Based on experimental measurements, the size of each local model is 3.712 Mbits.

All experiments were conducted using the PyTorch framework (version 2.5.1, with CUDA 11.8 support). The experiments were performed on a workstation equipped with an AMD Ryzen 9 5900X 12-core processor (Advanced Micro Devices, Santa Clara, CA, USA), 32 GB RAM, and an NVIDIA RTX 1660 GPU with 6 GB VRAM (NVIDIA Corporation, Santa Clara, CA, USA).

#### 5.1.2. Dataset

The dataset employed in the experiments is the public KuaiRec dataset [39], which was collected from the recommendation logs of the Kuaishou application. This real-world dataset comprises 7176 users, 10,728 videos, and 12,530,806 detailed interaction records. The input features for modeling user preferences include user ID, user activity level, encrypted user attributes (processed via one-hot encoding), video duration, watch time, and video category. The ground-truth labels are constructed based on user ratings for videos with observed interactions. Unless otherwise specified, we adopt a uniform data partitioning strategy to distribute the dataset across 10 local nodes, thereby simulating a federated learning environment. For each local node, the local dataset is further divided into 80% training, 10% validation, and 10% test sets.

#### 5.1.3. Baseline Methods

In this paper, several baseline methods are introduced to validate the effectiveness of the proposed federated learning framework.

To evaluate the generalization capability of the proposed bias-corrected local training scheme, the following widely used recommendation models are adopted:

- **FM** [10] is a classical model for capturing pairwise feature interactions in sparse settings. It has been widely applied in recommendation and click-through rate prediction tasks due to its effectiveness and computational efficiency.
- **DeepFM** [11] is a representative deep learning architecture that integrates Factorization Machines with a feed-forward neural network to jointly model low-order and high-order feature interactions. Specifically, DeepFM shares the same feature embeddings between the FM component, which captures second-order interactions, and the deep component, which learns nonlinear higher-order interactions in an end-to-end manner.
- **Wide&Deep** [12] is a hybrid framework that combines a linear (wide) model with a deep neural network to jointly learn memorization and generalization patterns. The wide component captures manually designed cross-feature interactions, while the deep component leverages dense embeddings to model high-order nonlinear feature interactions in an end-to-end fashion.

To verify the effectiveness of the proposed statistical aggregation scheme in the timeliness-aware federated learning scenario, the following federated optimization baselines are considered:

- **FedAvg** [16] is the standard baseline for federated optimization. In FedAvg, each client performs multiple steps of local stochastic gradient descent (SGD) on its private data, and the server aggregates the local model updates through weighted averaging to obtain the global model, enabling decentralized training without sharing raw data.
- **FedPAC** [20] is a personalized federated learning framework in which a shared feature extractor and collaboratively optimized classifiers are utilized to address data heterogeneity by aligning representations while preserving client-specific prediction layers.

### 5.2. Performance of Bias-Corrected Scheme

In the experiments, the proposed bias-correction method is implemented as an independent representation refinement stage prior to the training of downstream recommendation models. Specifically, we first obtain the initial user and video embeddings. Based on the cumulative gradient analysis, projection operations (Equations (26)–(28)) are then applied to remove duration-related and conformity-related components from the embedding space, resulting in intrinsic representations $Q_{m}^{\mathrm{int}}$ and $P_{s}^{\mathrm{int}}$. After this debiasing step, the corrected embeddings are treated as fixed input features for FM, DeepFM, and Wide&Deep. The recommendation models are subsequently trained using the standard binary cross-entropy loss for click-through rate prediction, without introducing additional projection or bias-removal operations during recommendation model optimization. Therefore, the debiasing procedure and the neural recommendation training are decoupled in our experimental pipeline. This design ensures that performance differences observed across models are attributable to the effectiveness of the bias-corrected representations rather than to joint optimization strategies or architectural modifications.

Table 1 reports the AUC and ACC performance of the DeepFM model under different settings of the bias-correction parameters $\alpha_{1}$ and $\alpha_{2}$, where AUC measures the probability that the model assigns a higher predicted score to a randomly selected positive sample than to a randomly selected negative sample and ACC measures the proportion of correctly classified samples among all samples. In addition, the two bias-correction parameters control the intensity of duration-bias removal in the video and user embeddings, respectively. When $\alpha_{1}=\alpha_{2}=0$, the original embedding vectors are directly used for model training, corresponding to the case without debiasing. In contrast, $\alpha_{1}=\alpha_{2}=1$ represents the strongest degree of bias correction. As shown in Table 1, both AUC and ACC gradually improve as $\alpha_{1}$ and $\alpha_{2}$ increase from 0 to moderate values. This improvement can be attributed to the removal of duration-related components from the embedding vectors, which enables the learned representations to better capture users’ intrinsic interest preferences and thus enhances prediction accuracy. However, when $\alpha_{1}$ and $\alpha_{2}$ are set to 1, a slight performance degradation is observed. This phenomenon arises because the duration-bias direction is approximated using the average embedding of long-duration videos, which cannot perfectly characterize the true duration bias. As a result, overly aggressive bias correction may inadvertently eliminate informative components that are beneficial for recommendation. Therefore, setting $\alpha_{1}=\alpha_{2}=0.9$ achieves a favorable trade-off, providing sufficient debiasing strength while preserving useful preference information in the embedding representations.

**Figure 5:**
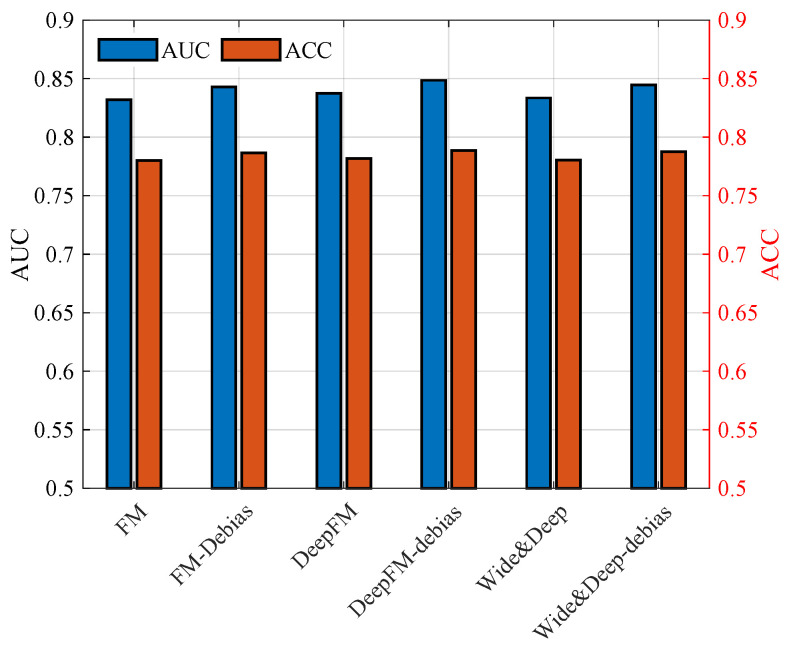
AUC and ACC performance among several classical recommendation models.

Figure 5 depicts the comparison of AUC and ACC performance among several classical recommendation models when trained with the original embedding vectors and the proposed debiased vectors. To ensure a fair evaluation of the debiasing effectiveness, the experimental configuration adopts the optimal parameter settings determined in Table 1. It is observed that all considered models achieve consistent performance improvements after applying the debiasing strategy. This improvement stems from the fact that the effectiveness of recommendation systems depends not only on data quality but also on the quality of feature representations. If inherent biases in the embeddings are not properly mitigated, the backpropagation process may further amplify such biases during training, thereby deteriorating the final recommendation performance. By contrast, removing the duration-related bias enables the models to learn representations that better reflect users’ intrinsic preferences, resulting in enhanced predictive accuracy.

### 5.3. Performance of Statistical Aggregation Scheme

**Figure 6:**
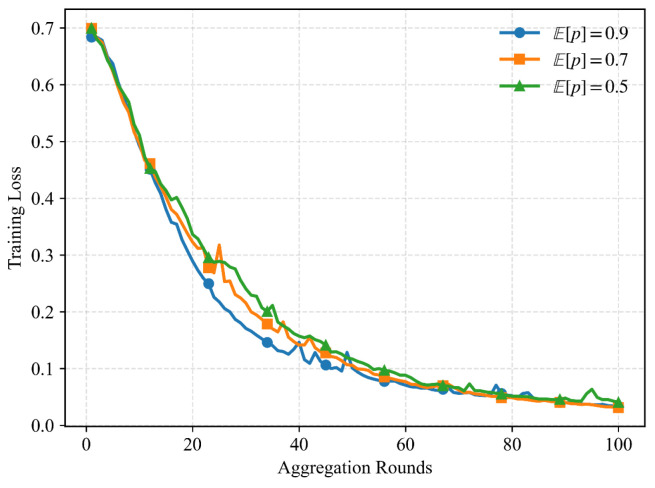
Training loss under different successful upload probabilities.

Figure 6 illustrates the evolution of the training loss under different successful upload probabilities. It can be observed that scenarios with higher upload success probabilities exhibit significantly faster convergence compared to those with lower success probabilities. This behavior can be explained by the fact that a higher successful upload probability allows more local model updates to be incorporated into the global aggregation at each communication round. Consequently, the global model benefits from richer and more diverse gradient information, leading to more efficient optimization and accelerated convergence. Additionally, despite the differences in convergence speed, the training losses under all considered cases eventually converge to similar values. This phenomenon demonstrates that the proposed statistical aggregation scheme effectively compensates for the randomness introduced by unreliable uploads. By correcting the aggregation weights according to the upload success probabilities, the proposed method ensures that the expected global update remains unbiased with respect to the ideal FedAvg aggregation under fully reliable communication. Therefore, while unsuccessful uploads may delay convergence, they do not compromise the final achievable performance, provided that a sufficient number of training rounds is conducted.

**Figure 7:**
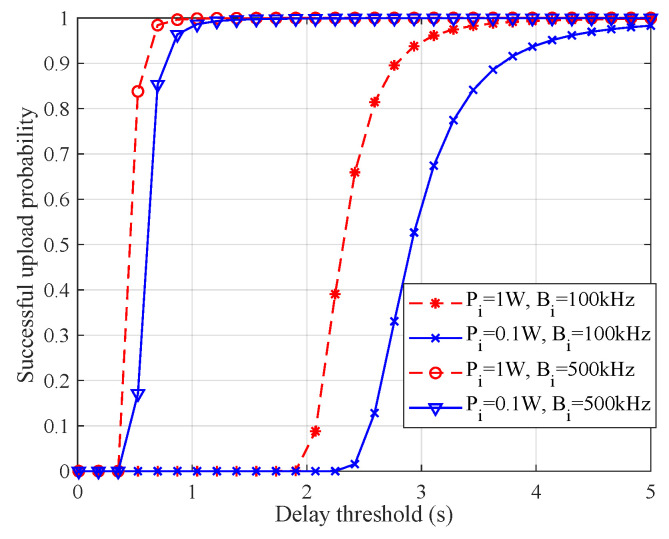
Successful upload probability varying with delay threshold.

To investigate the factors influencing the successful upload probability, Figure 7 illustrates the variation of the upload success probability with respect to the delay threshold for local model transmission. It can be observed that the successful upload probability increases as the delay threshold becomes less stringent. This is because a larger delay tolerance allows for more local nodes to complete model transmission within the permitted time window, thereby reducing the likelihood of upload failures. In addition, both the transmission power and the allocated bandwidth of local nodes have positive effects on the successful upload probability. Specifically, higher transmission power improves the received signal-to-interference-plus-noise ratio, while larger bandwidth enhances the achievable data rate. Both factors shorten the transmission time of local models, thus increasing the probability that uploads meet the delay constraint. These results indicate that the efficiency of federated learning can be improved not only by allocating more communication resources (e.g., power and bandwidth), but also by relaxing the timeliness requirement of model aggregation. However, loosening the delay constraint inevitably introduces additional latency into each aggregation round, potentially prolonging the overall training process. Therefore, a careful trade-off must be established between convergence speed and aggregation timeliness to achieve an efficient and practical federated learning deployment.

**Figure 8:**
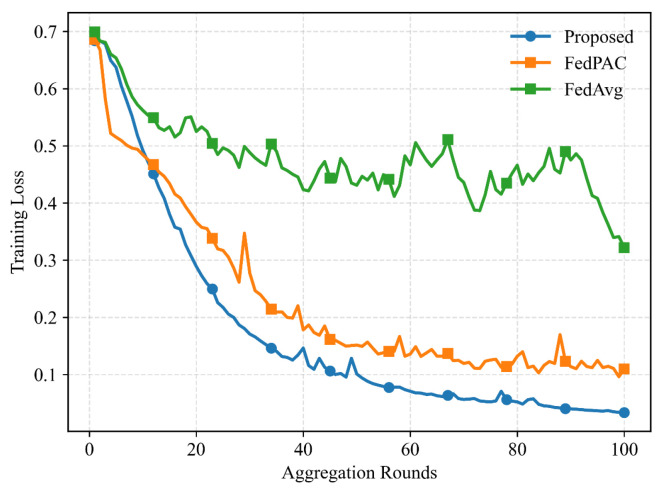
Training loss under different federated learning methods.

Figure 8 presents the training loss curves under different federated learning methods, where the input data for all three approaches are preprocessed using the proposed Bias-Corrected scheme. It can be observed that the proposed federated learning method consistently outperforms the two baseline schemes in terms of convergence behavior and final loss value. The performance degradation of FedAvg in this scenario can be attributed to the presence of unsuccessful model uploads. When upload failures occur, the conventional FedAvg aggregation becomes statistically biased relative to the ideal case with fully reliable communication, since the aggregation weights are implicitly affected by the random participation of local nodes. As a result, the global model update deviates from the optimal aggregation direction, leading to slower convergence and inferior performance. In addition, although FedPAC is designed to address data heterogeneity through personalized modeling, it does not explicitly account for communication unreliability during aggregation. Therefore, its performance is also influenced by stochastic upload failures, which introduce additional variance into the global update process. By contrast, the proposed method incorporates a statistically unbiased aggregation mechanism that explicitly compensates for the successful upload probability. This design effectively mitigates the deviation caused by unreliable communication, resulting in more stable convergence and improved overall performance.

**Figure 9:**
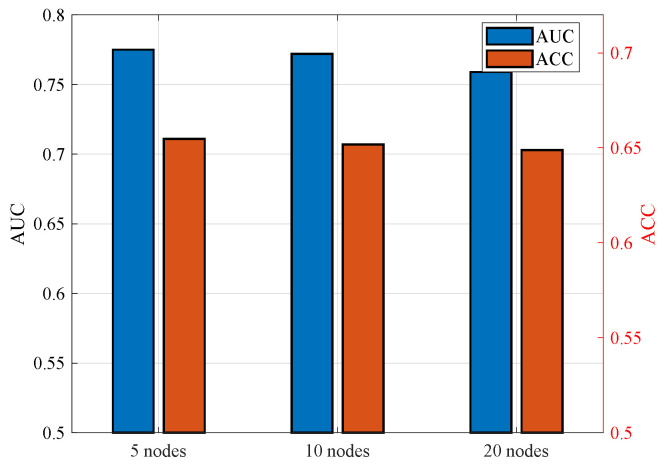
AUC and ACC Performance under different number of nodes.

Figure 9 illustrates the AUC and ACC performance under different numbers of participating local nodes. In this experiment, the total number of data samples is fixed and evenly distributed across all local nodes. Therefore, as the number of participating nodes increases, the number of data samples available at each node correspondingly decreases. It can be observed that scenarios with fewer participating nodes achieve higher AUC and ACC performance. This phenomenon can be explained from both the data distribution and communication perspectives. Under the default communication configuration, the successful upload probability is approximately 90%, which ensures that most local nodes can successfully transmit their model updates to the aggregator. When the number of nodes is small, each node possesses a larger portion of the overall dataset, enabling it to train a more representative and robust local model during each aggregation round. Consequently, the aggregated global model benefits from higher-quality local updates, resulting in superior overall performance. In contrast, when the number of nodes increases, each node is assigned fewer training samples. Although more nodes participate in the aggregation, the reduced data volume per node may limit the quality of local model training, thereby slightly degrading the final recommendation performance. This result highlights the trade-off between local node granularity and local training effectiveness in federated learning systems.

**Figure 10:**
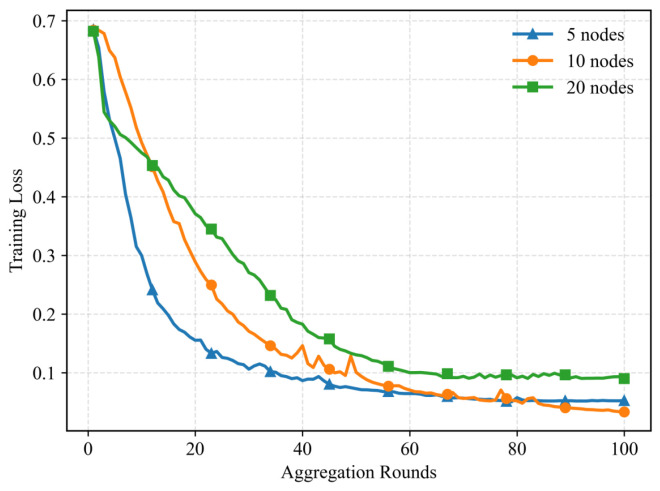
Training loss under different node configurations.

To evaluate the convergence behavior under dynamic participation, Figure 10 illustrates the evolution of training loss with varying numbers of participating nodes. It is observed that the configuration with fewer nodes (5 nodes) achieves a faster convergence rate and reaches a lower training loss compared to scenarios with more nodes. This phenomenon aligns with the performance trends shown in Figure 9. When the total dataset size is fixed, fewer participating nodes imply that each node possesses a larger portion of data, enabling the training of more representative local models. Consequently, the global model benefits from higher-quality updates, leading to more stable and efficient convergence. Despite the slight degradation in convergence speed with increased node counts (due to reduced local data volume per node), the proposed method maintains stable training trajectories across all settings, demonstrating its robustness in dynamic federated learning environments.

### 5.4. Performance Among Non-IID Scenarios

To evaluate the robustness of the proposed method under heterogeneous data distributions, we extend the experimental setup from the standard IID partition to two non-IID scenarios: duration-based partition and activity-based partition. The duration-based setting groups users according to their average viewing duration and watch ratio, while the activity-based setting categorizes users by interaction frequency. These settings simulate practical distribution shifts caused by preference bias and activity imbalance in federated recommendation systems.

As reported in Table 2, data heterogeneity consistently degrades the performance of FedAvg across all metrics. Under the IID setting, FedAvg achieves an AUC of 0.6821 and an HR@10 of 0.7332, which decrease to 0.6443/0.6801 in the duration-based scenario and 0.6414/0.6386 in the activity-based scenario. Similar degradation is observed for ACC, NDCG@10, and Recall@10, confirming the adverse effect of non-IID data on federated optimization. In contrast, the proposed method consistently achieves the best performance across all data distributions. Under the IID setting, it improves AUC from 0.6821 to 0.7712 and HR@10 from 0.7332 to 0.8086. More importantly, the performance decline under non-IID scenarios is significantly alleviated. For example, under the duration-based setting, the proposed method maintains an AUC of 0.7444 and an HR@10 of 0.7872, substantially outperforming both FedAvg and FedAvg-Debias. Similar advantages are observed under the activity-based scenario. Consistent improvements are also reflected in ranking metrics, including NDCG@10 and Recall@10. Overall, the results demonstrate that the proposed method enhances both classification performance (AUC, ACC) and ranking quality under heterogeneous federated settings, validating its effectiveness in mitigating the impact of data heterogeneity.

## 6. Conclusions

In this paper, we studied the problem of real-time video recommendation in distributed wireless environments and developed a bias-aware federated learning framework to address both learning and communication challenges. By explicitly considering the impact of video duration and user activity, the proposed method alleviates representation bias during local training and enables the global model to better capture users’ intrinsic preferences. In addition, by modeling the successful upload probability under stochastic communication links, we designed a statistically robust aggregation strategy that preserves the unbiasedness of the global update even in the presence of delayed or failed transmissions. Experimental evaluations verified that the proposed approach not only enhances recommendation accuracy, but also maintains stable convergence performance under unreliable communication conditions. Overall, this work provides an effective and practical solution for deploying federated video recommendation systems in dynamic and resource-constrained network environments.

## Figures and Tables

**Table 1:** Performance under different debias levels.

Parameter Settings		Performance	
$\alpha_{1}$	$\alpha_{2}$	ACC	AUC
0	0	0.7818	0.8375
0.3	0.3	0.7861	0.8426
0.5	0.5	0.7887	0.8461
0.9	0.9	0.7909	0.8486
1.0	1.0	0.7901	0.8481

**Table 2:** Performance comparison under different data distributions.

Methods	Data Distribution	AUC	ACC	NDCG@10	HR@10	Recall@10
FedAvg	IID	0.6821	0.5432	0.5486	0.7332	0.6622
	Duration-based	0.6443	0.5012	0.4891	0.6801	0.6013
	Activity-based	0.6414	0.5179	0.4907	0.6386	0.5761
FedAvg-Debias	IID	0.7331	0.6171	0.5634	0.7593	0.6931
	Duration-based	0.6931	0.5822	0.5177	0.7327	0.6514
	Activity-based	0.6914	0.5776	0.5123	0.7346	0.6461
Proposed	IID	0.7712	0.6521	0.5844	0.8086	0.7152
	Duration-based	0.7444	0.6311	0.5418	0.7872	0.6743
	Activity-based	0.7402	0.6395	0.5541	0.7857	0.6678

## Data Availability

The original data presented in the study are openly available in KuaiRec: A Fully-Observed Dataset and Insights for Evaluating 589 Recommender Systems at https://kuairec.com/; https://hexiangnan.github.io/papers/cikm22-kuairec.pdf, accessed on 6 April 2026.
